# RNA Whole-Mount *In situ* Hybridisation Proximity Ligation Assay (rISH-PLA), an Assay for Detecting RNA-Protein Complexes *in Intact Cells*

**DOI:** 10.1371/journal.pone.0147967

**Published:** 2016-01-29

**Authors:** Ioannis M. Roussis, Matthew Guille, Fiona A. Myers, Garry P. Scarlett

**Affiliations:** Biophysics Laboratories, Institute of Biomedical and Biomolecular Sciences, University of Portsmouth, Portsmouth, PO1 2DT, United Kingdom; Xuzhou Medical College, CHINA

## Abstract

Techniques for studying RNA-protein interactions have lagged behind those for DNA-protein complexes as a consequence of the complexities associated with working with RNA. Here we present a method for the modification of the existing *In Situ* Hybridisation–Proximity Ligation Assay (ISH-PLA) protocol to adapt it to the study of RNA regulation (rISH-PLA). As proof of principle we used the well-characterised interaction of the *Xenopus laevis* Staufen RNA binding protein with Vg1 mRNA, the complex of which co-localises to the vegetal pole of *Xenopus* oocytes. The applicability of both the Stau1 antibody and the Locked Nucleic Acid probe (LNA) recognising Vg1 mRNA were independently validated by whole-mount Immunohistochemistry and whole-mount *in situ* hybridisation assays respectively prior to combining them in the rISH-PLA assay. The rISH-PLA assay allows the identification of a given RNA-protein complex at subcellular and single cell resolution, thus avoiding the lack of spatial resolution and sensitivity associated with assaying heterogenous cell populations from which conventional RNA-protein interaction detection techniques suffer. This technique will be particularly usefully for studying the activity of RNA binding proteins (RBPs) in complex mixtures of cells, for example tissue sections or whole embryos.

## Introduction

Although there is increasing interest in post-transcriptional gene regulation and RNA modulation [1,2], the technologies available to assay RNA-Protein interactions have lagged behind those for DNA-Protein interactions, as a consequence of the difficulties associated with working with cellular RNAs.

**Fig 1:**
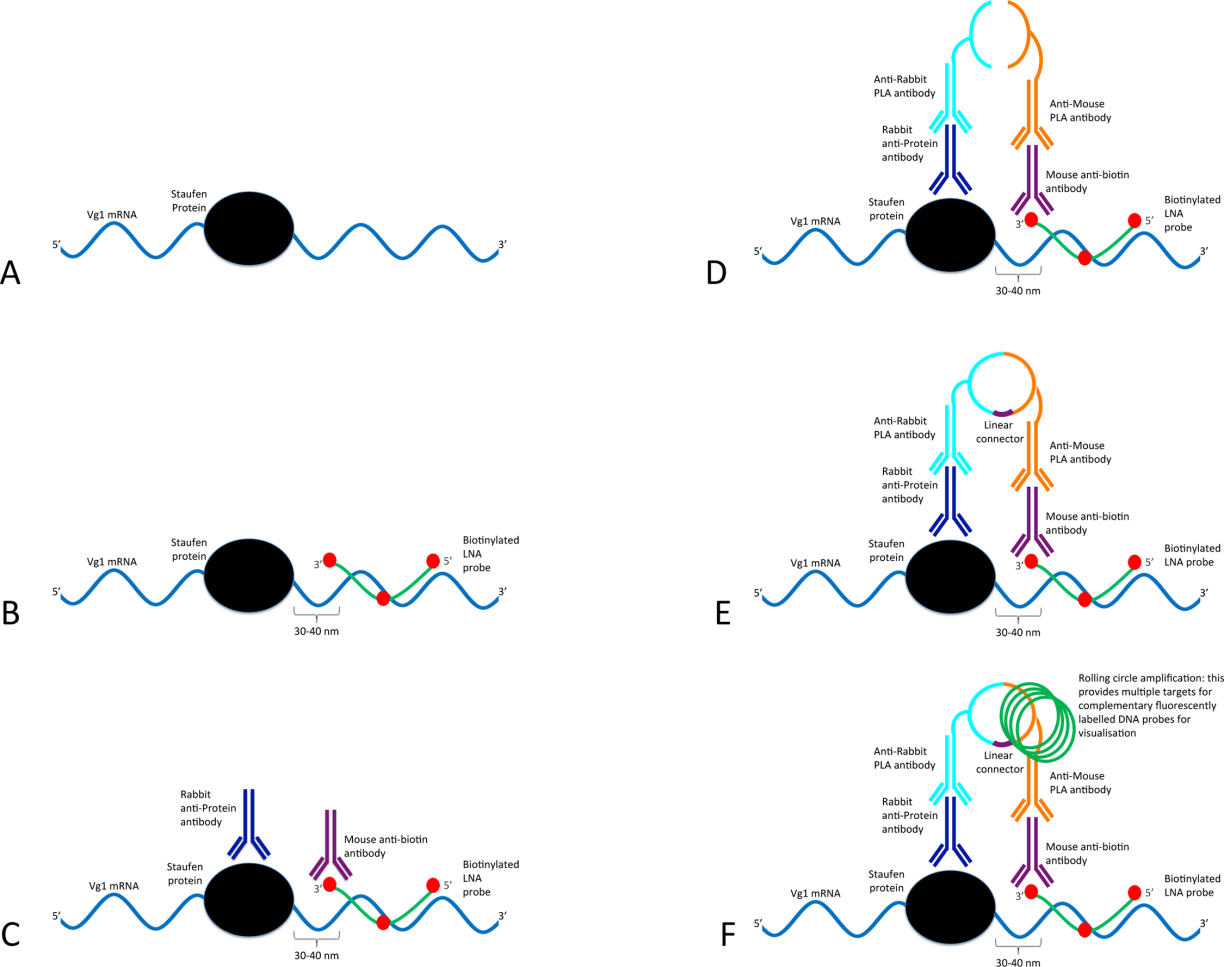
Schematic of the ISH-PLA modified for use with RNA (rISH-PLA). The ISH-PLA technique has been developed for the analysis of the co-localisation of protein complexes and specific DNA sequences. In this paper the method is adapted for RNA. **(A)** *In vivo* interaction of Vg1 mRNA (blue) with *Xenopus* Staufen (black)**. (B)** In the first step a biotinylated LNA probe (green with biotin indicated in red) targets a specific RNA sequence close to the binding site of XStau1 in the Vg1 mRNA is added (see results section for details on LNA probe design). **(C)** Two primary antibodies raised in different species are added. In this case a rabbit antibody (dark blue) targets the XStau1 and a mouse antibody (purple) targets the biotin label in the LNA probe. **(D)** Two secondary PLA species-specific antibodies (light blue and orange) conjugated to oligonucleotides are then added. Since the LNA probe and the protein are in close proximity the secondary antibodies and their conjugated oligonucleotides are consequently also in close proximity. **(E)** The conjugated oligonucleotides are joined and circularised by DNA ligation upon introduction of linear connector oligonucleotide (dark purple). **(F)** The two oligonucleotides then commence rolling circle replication (the amplified circular DNA molecule is annotated in green). After the amplification reaction, fluorescent labelled complementary oligonucleotide probes are added to highlight the product.

*In-situ* hybridisation Proximity Ligation Assay (ISH-PLA) (Fig 1) is a relatively new technique developed at the University of Virginia [3] that enables the visualisation of the co-localisation of proteins and defined DNA sequences with single cell resolution. This technique has been used to great effect in investigating mechanisms of DNA-protein interactions that contribute to gene regulation, highlighted by a number of reports of its use in epigenetic analysis [3–5]. ISH-PLA combines *in situ* hybridisation (ISH) with the proximity ligation assay (PLA) and one of its first achievements show that di-methylation of lysine 4 of histone H3 (H3K4me2) at the MYH11 locus is restricted to the smooth muscle cell (SMC) lineage in human and mouse tissue sections. Further, it showed that this mark persisted even in phenotypically modulated SMC present in atherosclerotic lesions that show no detectable expression of SMC marker genes [3]. The main advantage of ISH-PLA over existing chromatin immunoprecipitation technologies is the ability to identify interactions at the single cell level in a heterogeneous sample.

In order to study cells of a specific type in their normal environment there is a need to use techniques to purify them, which often involves generating transgenic lines of animals [6]. Alternatively assays that can work on fixed, whole embryos or tissue sections at single cell resolution like ISH-PLA can be utilised. The requirement for a technique of this latter type to study RNA-protein interactions is very necessary. Currently the most commonly used techniques for RNA-protein interactions are either the RNA immunoprecipitation assay (RIP), which is very prone to false positives [7], or the more sophisticated but far more technically demanding cross-linking immunoprecipitation (CLIP) assay [8,9]. Both of these techniques assay RNA binding activity in total cellular extract, meaning that data from a heterogeneous sample can be lost due to a dilution of the positive signal or may include false positives generated by interactions that occur in contaminating cells. In addition to distinguishing between individual cells the ISH-PLA technique can pinpoint interactions at a sub-cellular level [10] and such data are likely to be very important for understanding the function of RNA-protein interactions. We therefore decided to adapt the ISH-PLA technology for use in RNA studies (rISH-PLA).

Initially in the ISH-PLA assay a nucleic acid probe targets a specific genomic sequence close to the binding site of the cognate binding protein. Two primary antibodies raised in different species are added, one (raised in rabbit) targets the cognate protein and a second antibody (raised in mouse) targets the biotinylated nucleic acid probe. Subsequently two secondary species-specific PLA antibodies with conjugated PLA oligonucleotides are added. Since the probe and the cognate binding protein are in close proximity the conjugated oligonucleotides are also in close proximity and are circularised upon introduction of a linear connector oligonucleotide, allowing rolling circle replication to commence. After the amplification reaction, far-red fluorescently labelled complementary oligonucleotide probes are added to highlight the product. Normally in ISH-PLA the nucleic acid probe binding to the target gene is around 500 bp in length, however since mature messenger RNA transcripts are generally in the several kilobase range this length probe would lack resolution in terms of determining the site bound by the RNA binding protein. Further, RNA may adopt a wide range of secondary and tertiary structures which can interfere with probe binding [11,12]. Together these mean that there is a need to develop a protocol specifically for use with RNA.

To develop the RNA based ISH-PLA we have exploited the well-established interaction of *Xenopus* staufen (Stau1) with Vg1 mRNA, the complex of which localises to the vegetal pole in oocytes (Young et al. 2004). Staufen1 is a double stranded RNA binding protein (dsRBP) [13] which binds to vegetally localized mRNA to form a ribonucleoprotein complex and is also associated with other proteins such as: kinesin motor proteins, ribosomal P-stalk protein P0, protein phosphatase 1, nucleolin, β4-tubulin, NFAR, hnRNPU, RNA helicase A, neuronal protein Sharpin and Prrp amongst others (Brendel et al., 2004). Vg1 RNA is localized in a tight crescent at the vegetal pole of mature oocytes, i.e., in the cytoplasmic region that will become the endoderm [14]. The Vg1 mRNA encodes a member of the transforming growth factor-β superfamily implicated in giving endodermal cells special properties such as the capacity to induce mesoderm (mesoderm formation) and establishment of the left and right asymmetry in the developing embryo [15–17]. Stau1 interacts with the Vg1 mRNA sequence through two repetitive motifs (VM1 and E2), found in a 340 nucleotide cis-acting localization signal contained within the 3’ UTR of the Vg1 mRNA, which possesses extensive secondary structure [18]. Both motifs are mandatory for vegetal transport [19]. An advantage of using the oocyte for the development of this assay is the fact that it has historically been a difficult cell to work with in terms of immunohistochemistry, requiring significant technique optimisation [20], thus there is a good chance that technique will be transferable to other cells.

There is a clear need for a sensitive assay for RNA-protein interactions that permits subcellular and single cell resolution analysis in a variety of samples including tissue sections and whole-mounts. Here we describe the modifications necessary to adapt the existing DNA based ISH-PLA technique to mRNA.

## Materials and Methods

### Western Blot

Embryo protein lysate was extracted in embryo extraction buffer (10 mM HEPES (pH 8.5), 2 mM MgCl_2_, 1 mM DTT, 1 mM EDTA, protease inhibitor cocktail tablet (Roche)) and one embryo equivalent resolved by SDS-PAGE gel electrophoresis and electro-blotted onto a nitrocellulose blotting membrane (Life Sciences). The membrane was blocked and probed following standard protocols [21], with the rabbit anti-XStau1 antibody at a 1:10,000 dilution.

### Animal Care and Husbandry

Adult female *Xenopus laevis* were obtained from the European *Xenopus* Resource Centre (EXRC) at the University of Portsmouth and maintained at 18.5C in recirculating water with 10% daily changes. They were fed 3–5 times daily on a high protein pelleted diet. All animal care and experimentation was approved by the University of Portsmouth AWERB and performed under ASPA (1986) licences. This work, licensed under project number 70/7661, complies with Home Office regulations regarding the use of animals in scientific research. All surgery was performed under sodium pentobarbital anesthesia and all efforts were made to minimize suffering.

### Whole-mount Immunohistochemistry

Ovarian lobes were removed from *Xenopus laevis* females and oocytes separated by collagenase treatment and staged [22]. Oocytes were fixed using 4% paraformaldehyde at room temperature for a minimum of 2 hours. After stepwise rehydration in PBS containing 0.05% Triton X-100, stage III–IV oocytes were washed eight times for 5 minutes each with PBS/0.35% Triton X-100 prior to rinsing in PBS and then blocked for at least 2 h in 1% BSA/PBS at 4°C. Samples were incubated with anti-XStau1 antibody (kind gift of N. Standart) overnight at 4°C and subsequently secondary antibody (Alexa Fluor® 647 donkey anti-rabbit IgG from Molecular Probes, A31572) for 2 hours at room temperature. Finally the oocytes were washed in PBS/0.1% Tween-20 and mounted in 100% Glycerol mounting media. Images were acquired using confocal microscopy (Zeiss LSM510) at 644 nm.

### Molecular Biology and cloning

RNA was extracted from stage III/IV *Xenopus laevis* oocytes [23] and reverse transcribed to a cDNA library (Precision nanoScript^TM^ 2 Primerdesign). Forward (AATATGTGTGCTACAGATCC) and reverse (TGACCTGTTAGATCTATCACT) primers were used to PCR amplify a 480-bp of the Vg1 3’UTR from the library (annealing 58°C, 30 sec; extension 72°C, 30 sec; 35 cycles) and the product subcloned into the pCR®2.1-TOPO TA vector (TOPO TA Cloning®, Invitrogen), putative clones were confirmed by Sanger sequencing (Source Bioscience). The confirmed clone was then subcloned into pBluescript II between SacI and NotI restriction sites 5’ and 3’ respectively. RNA was *in vitro* transcribed from the construct using MEGAascript (Ambion).

### Electrophoretic Mobility Shift Assay

Either the DNA analogue of the proposed LNA probe (Invitrogen) or the *in vitro* transcribed 480-bp 3’UTR of the Vg1 mRNA was labelled and analysed by EMSA which was conducted as described previously [24]. The nucleic acid was mixed with one embryos equivalent of total protein extract per lane in the presence of competitors and separated on a gradient Mini-PROTEAN®TGX^TM^ Precast Gel from BIO-RAD. The samples were run at 20 mA for 75 min at 4°C.

### Whole-mount in-situ hybridisation (WISH)

Whole-mount *in-situ* hybridisation was performed on stage III-IV *Xenopus laevis* oocytes following the protocol described previously [23] with the following changes. The bleach step was not performed in order to allow for the orientation of the oocytes. In the pre-hybridisation step, the oocytes were incubated with the *in situ* hybridisation buffer for 30 minutes at 70°C and 5.5 hours at 60°C. Finally for the probe visualisation, a fluorescent antibody (Goat Anti-Mouse IgG Alexa fluor® 647, ab150115) was used at a 1:100 dilution instead of an anti-digoxygenin antibody.

### Whole-mount in-situ hybridisation proximity ligation assay (WISH-PLA)

A full protocol is provided in S1 Text. The WISH protocol as described above was performed up to the 5 hour blocking step with the MAB / 2% Block before the incubation with the primary antibody followed directly by 1 hour blocking with agitation in the PLA blocking solution (Duolink®). After the blocking step, whole-mount samples were incubated with rabbit anti-XStau1 (2 μg/ml) and mouse anti-biotin (2 μg/ml, ab201341, Abcam) antibodies overnight at 4°C, followed by incubation with secondary antibodies conjugated with PLA probe at 37°C for 2.5 h. The ligation (1 h) and amplification (200 minutes) steps were performed with agitation at 37°C (Duolink detection kit Far Red, 644 nm). Oocytes were held in place for confocal microscopy with 100% Glycerol mounting media.

## Results and Discussion

### The Xenopus anti-Staufen antibody and the LNA probe both work when tested independently

**Fig 2:**
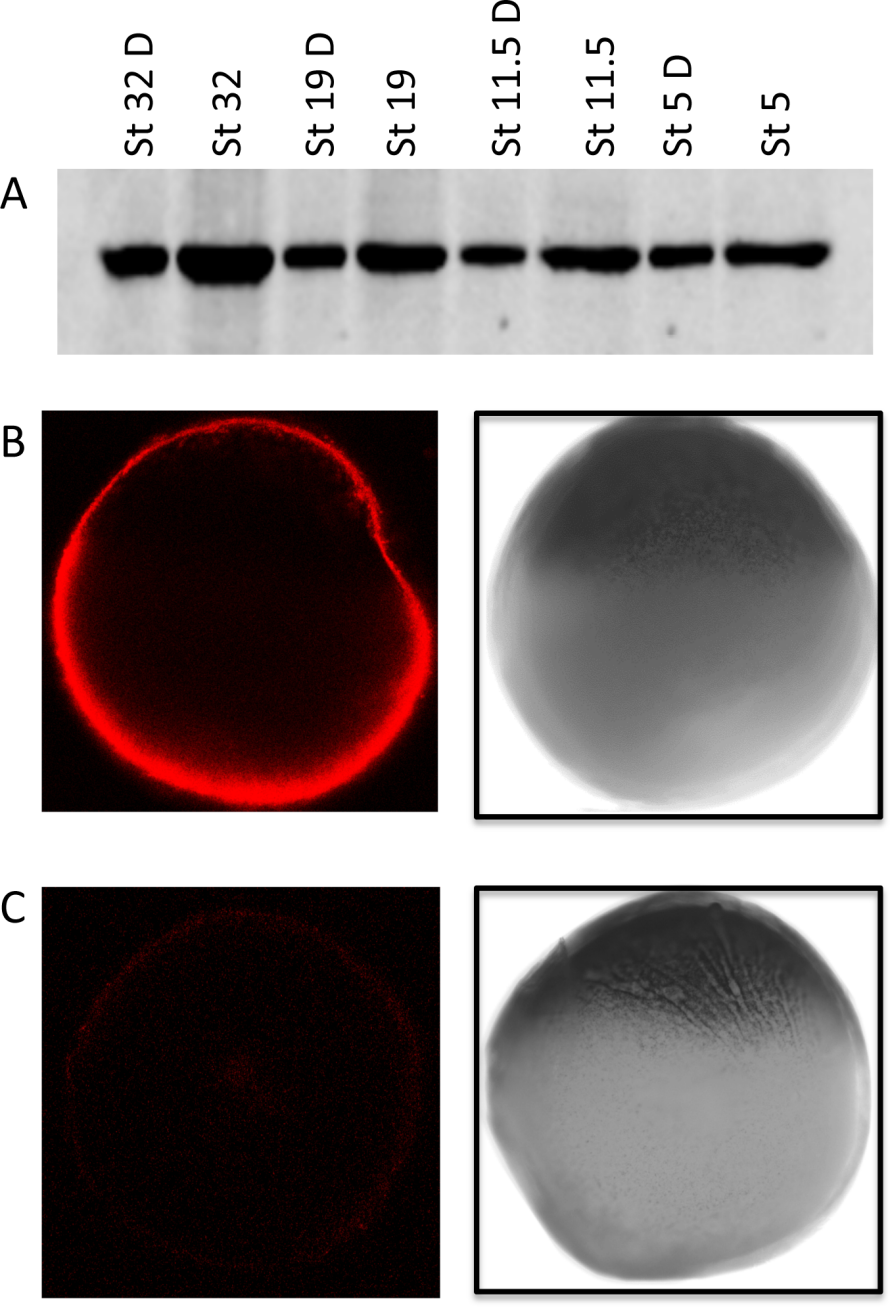
Western Blot and Whole-mount Immunohistochemistry using rabbit anti-Stau1 antibody. (A) Protein was prepared from *Xenopus laevis* embryos at stages 5–32 and yolk removed from the extraction; protein corresponding to one or a half (D) oocyte equivalents was separated by 12% SDS-PAGE and transferred to a nitrocellulose membrane prior to probing with a staufen specific antibody. A single, strong band of 80 kDa, corresponding to the molecular weight of staufen was detected. (B) Whole-mount Immunohistochemistry was performed on *Xenopus laevis* oocytes stage IV and V using the anti-Staufen antibody. The oocytes were visualised with Alexa Fluor® 647 donkey anti-rabbit antibody, which recognises the rabbit anti-XStau1 antibody, and photographed by confocal microscopy at 644 nm (left panel) and also by light microscopy (right panel) to identify the vegetal pole. (C) No signal was detected in the control samples where no primary antibody was present; the confocal image is shown on the left panel and light microscopy image on the right.

As a positive control for the RNA based ISH-PLA we used the well-established interaction of *Xenopus* staufen (Stau1) with Vg1 mRNA, the complex of which localises to the vegetal pole in oocytes. Initially a rabbit anti *Xenopus* Stau1 antibody (a kind gift from Dr Nancy Standart, University of Cambridge), was assayed for its specificity and applicability to *in-situ* hybridisation experiments. This was first tested by Western blot analysis, Fig 2A shows probing of *Xenopus laevis* total protein extract at a range of developmental stages with the anti-XStau1 antibody. The antibody is specific to a single protein corresponding to a molecular weight of 80 kDa, the expected size of *Xenopus* Staufen. Given the propensity of antibodies to recognize their epitopes when in the exposed state of a Western blot but not when in a complete *in vivo* complex we also tested the Stau1 antibody as the primary antibody in a whole-mount Immunohistochemistry assay (Fig 2B) whose analysis shows that the signal is specifically localized to the vegetal pole as expected for Stau1 in 9 out of 10 of the oocytes analysed. These results are in agreement with previously published data [25] and confirm that the antibody is sufficiently specific for rISH-PLA.

Since mRNA is much shorter than genomic DNA, the probes required for rISH-PLA must correspondingly be smaller to obtain a reasonable resolution. As a consequence it was decided to use a Locked nucleic acid (LNA) probe rather than a DNA or RNA probe due to the improved specificity and sensitivity of LNAs [11,12,26], which enables the design of a much shorter probe (20–30 bp). This is an important consideration when determining the localisation of the protein binding protein site on the target sequence on the mRNA probes. LNA modified nucleic acid probes have previously been widely used in *in situ* assays [27–29] and consist of RNA monomers with a modified backbone, in which the sugar phosphate backbone has a 2’-O-4’-C methylene bridge. The methylene bridge locks the nucleic acid probe into the N conformation which pre-organises the structure of the probe based on its complementary RNA target, allowing a more specific and stable binding. The design and efficacy of the LNA probe is therefore crucial to successful rISH-PLA.

**Fig 3:**
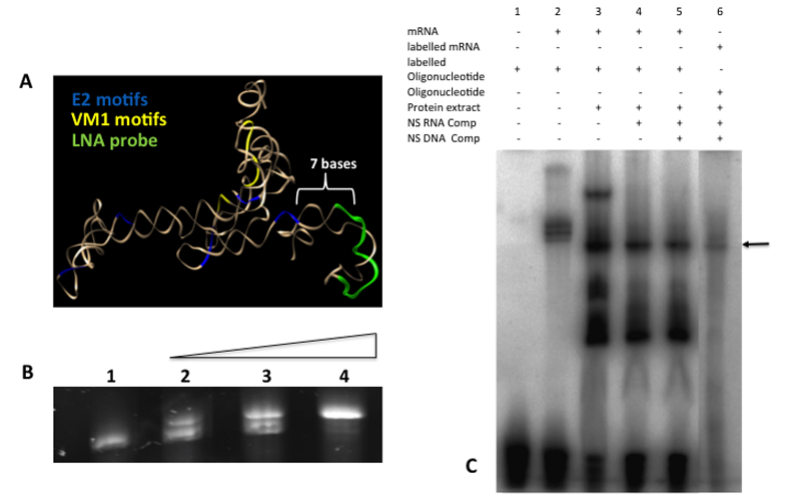
The proposed probe sequence can interact with both Vg1 mRNA and staufen protein. (A) The predicted 3D structure of the Vg1 mRNA 3’ UTR with the E2 motifs (blue), VM1 motifs (yellow) highlighted. The LNA probe (green) was designed to hybridise seven bases away from the last 3’ E2 motif, in close proximity to the predicted binding site of the protein complex that includes the Stau1 protein. (B) Syber gold stained EMSA, lane 1 control 3’UTR Vg1 mRNA (60nM). Lanes 2–5, increasing titration of the probe sequence DNA analogue: 30, 60 and 300 nM. (C) EMSA using acrylamide gel analysis. Lane 1, radiolabelled DNA probe sequence alone; lane 2–5 contain probe and 600 nM *in vitro* transcribed Vg1 3’UTR; Lane 3 in addition contains one oocyte equivalent of protein extract; lanes 4 and 5 are as lane 3 but with the addition of RNA and DNA non-specific competitors respectively. Lane 6 contains radiolabelled *in vitro* transcribed Vg1 3’ UTR in the presence of unlabelled probe sequence and one oocyte equivalent total protein extract along with DNA and RNA competitors.

Staufen does not bind to the entire Vg1 mRNA sequence but rather to two repetitive motifs (VM1 and E2) found in the 3’ UTR of the Vg1 mRNA that has extensive secondary structure. Primary considerations of the probe design are; (1) The LNA probe must be in close proximity (30–40 nm) to the binding site of XStau1 to Vg1 mRNA; it is important to note that in a highly structured RNA molecule sequences that are far apart in the primary sequence may be close in three dimensional space. (2) That the probe is targeting an exposed region of the RNA; if the RNA target sequence is already directly in intra-molecular or inter-molecular interactions then probe binding will be inhibited. Both of these considerations require an understanding of the predicted tertiary structure of the RNA. To this end the secondary structure of the 3’ UTR region (bases 1296 and 1776 of the fully transcribed unit of the *Xenopus laevis* Vg1 mRNA, accession number: NM-001095591.1) was initially predicted by Mfold (http://unafold.rna.albany.edu/?q=mfold). This information was then fed into RNAcomposer, an automated RNA structure 3D Modeling Server (http://rnacomposer.cs.put.poznan.pl/), to provide a tertiary structure (Fig 3A). Based on the predicted tertiary structure of the Vg1 3’ UTR the LNA probe was designed seven base pairs away from the last repetitive 3’ motif (E2). This predicted that the LNA probe would be in close proximity to the binding site of XStau1 to Vg1 mRNA; this is essential to allow rolling circle replication that is critical for signal detection to occur [26]. The selected region was also not predicted to be involved in intramolecular base pairing.

### The Staufen/Vg1 3’UTR complex is not disrupted by probe binding

To confirm the interaction of the proposed LNA probe sequence and the 3’ Vg1 UTR experimentally the sequence was first ordered as a DNA oligonucleotide analogue (due to cost considerations since several probes may need to be tested) and an electrophoretic mobility shift assay (EMSA) was performed (Fig 3B and 3C). Initially an *in vitro* transcribed 480 bases of the 3’ Vg1 UTR was incubated with increasing concentrations of the DNA analogue of the probe (Fig 3B); a small but significant shift was observed, demonstrating that the selected probe sequence was able bind to the *in vitro* transcribed 3’ UTR of the Vg1 mRNA, at least in the absence of Stau1 or any other protein components of the RNP complex.

To check for the ability of the probe to bind in the presence of the protein complex a second EMSA was performed on a native gradient gel (4–20%). The interaction between the labeled DNA analogue of the LNA probe (referred to as “probe” hereafter) and the Stau1 3’ UTR was confirmed (Fig 3C, compare lanes 1 and 2). A shift in the 3’ UTR-probe complex occurred when oocyte protein was added to the reaction (arrowed in lane 3 Fig 3); this must still contain the probe since it is the sole labeled component in the reaction. The mobility shift, together with retention of label, strongly support the existence of a stable ternary complex between the 3’UTR, an oocyte protein and the probe. Direct labeling of the 3’UTR sequence (lane 6) confirmed that the complex visualized by labeling the probe has the same mobility as the well-characterized vg1 3’UTR/Stau1 complex [13,30].

**Fig 4:**
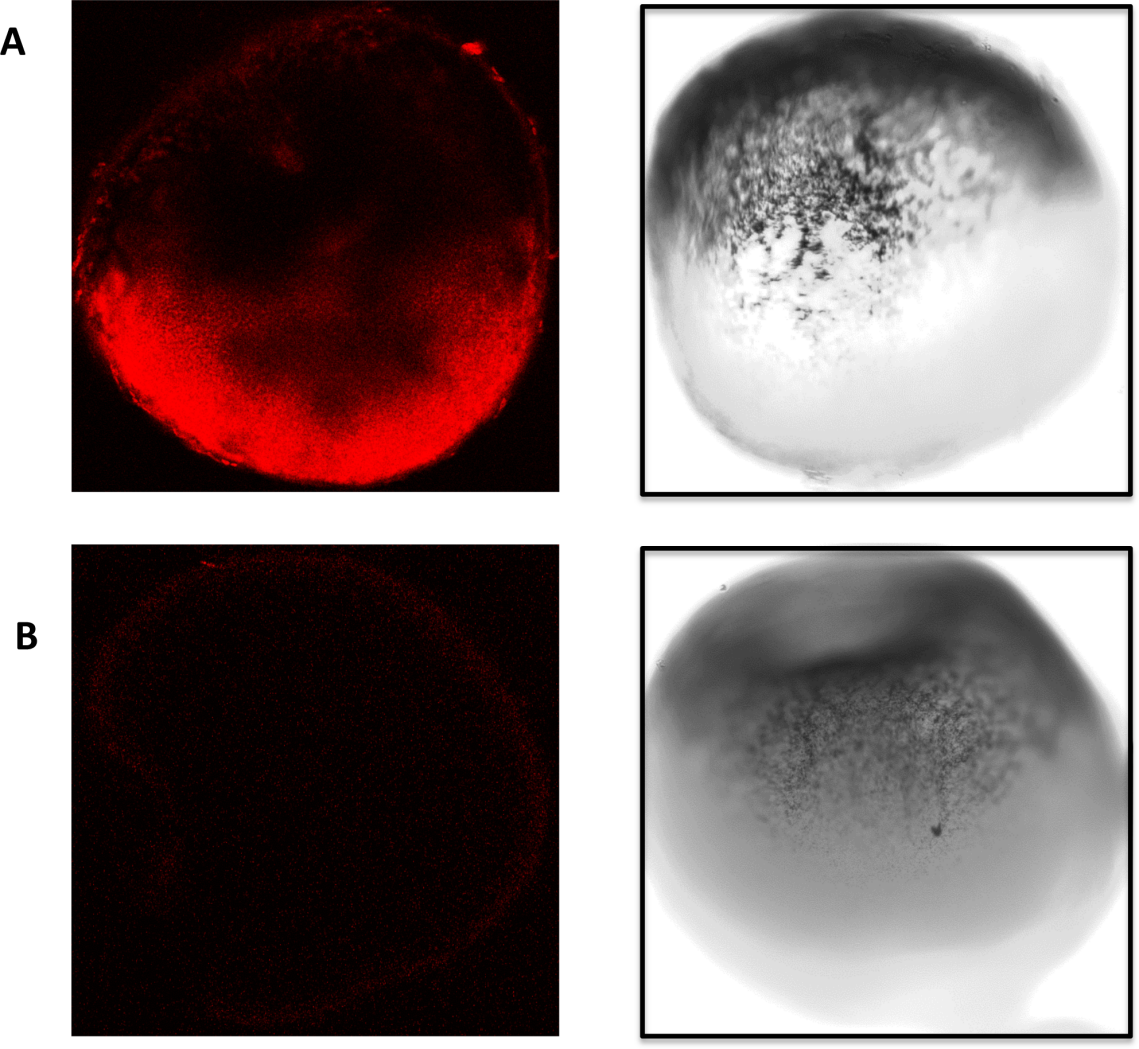
WISH using the biotinylated LNA probe in stage IV-V oocytes. (A) Whole-mount *in-situ* hybridisation was performed in *Xenopus laevis* stage IV and V oocytes. The oocytes were stained with an Alexa Fluor® 647 goat anti-mouse antibody which recognises the mouse anti-biotin antibody which recognises the biotin in the LNA probe. Confocal images at 644 nm are shown (left panel) alongside light microscopy images (right panel). (B) No signal was detected in the control samples when no primary antibody was used. Again confocal images are presented to the left and light microscopy images on the right.

Having confirmed that the DNA analogue of the proposed LNA probe was able to interact with Vg1 encoding mRNA in the presence of the Staufen protein the probe sequence was synthesised as a biotinylated LNA oligonucleotide. This probe was next tested in preliminary whole-mount *in-situ* hybridisation assay (WISH) conducted to optimize the protocol prior to extending to the rISH-PLA methodology. Fig 4 shows a localised signal at the vegetal pole of the *Xenopus laevis* oocytes in 10 out of 10 of the stage IV and V oocytes analysed. Our results are in agreement with previously published data [25], which also show a localisation of Vg1 mRNA to the vegetal pole of the oocyte and together with the EMSA confirm the suitability of the probe for use in the rISH-PLA.

### rISH-PLA reveals the staufen/vg1 mRNA complex in whole oocytes

**Fig 5:**
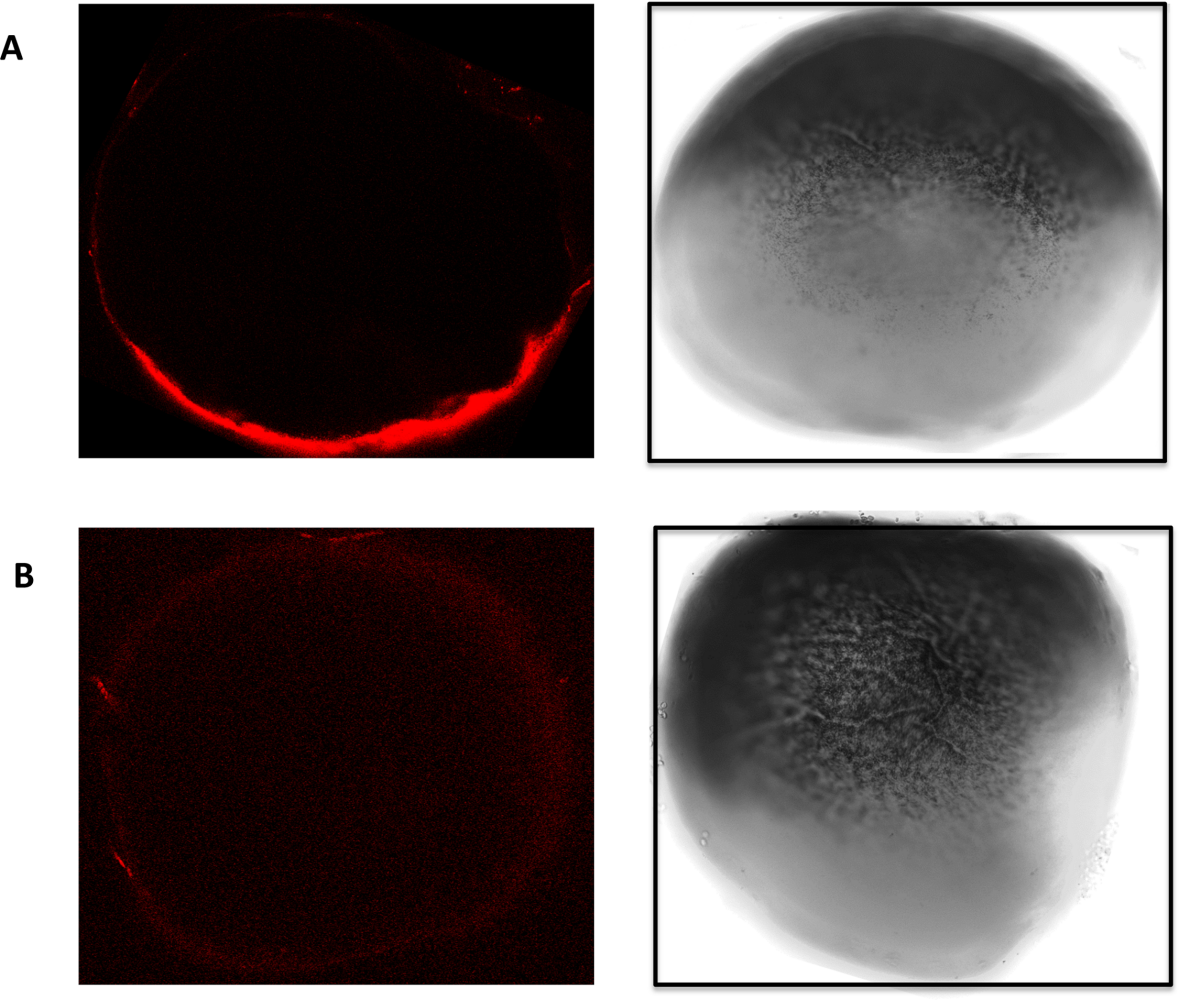
rISH-PLA assay for RNA analysis in stage IV-V oocytes. A. Whole-mount *in-situ* hybridisation proximity ligation assay was performed on *Xenopus laevis* stage IV and V oocytes. The oocytes where visualised by confocal microscopy at 644 nm, which shows localisation of Vg1-XStau1 complex in the vegetal pole of the oocytes. B. The control oocytes were incubated with an alternative primary antibody (rabbit anti AcH4) to assess specificity. No fluorescence is detected in the control samples (lower panel).

After the successful testing and optimization of the whole-mount immunohistochemistry and WISH assay, these two assays were merged along with the proximity ligation assay (PLA) to assess and optimise the ISH-PLA assay for RNA analysis. Two separate experiments were performed, each using 10 oocytes. In 70% (14/20) of the tested oocytes the observed signal pattern generated by rISH-PLA (Fig 5) was strong and identical to the superimposition of the signal pattern of the whole-mount immunohistochemistry assay onto the WISH assay (Figs 2C and 4). Fluorescence is only in the area where the Stau1-Vg1 mRNA complex is formed. As a control for the specificity of the WISH-PLA assay for RNA analysis, the protocol was performed identically with the exception that the rabbit anti-XStau1 antibody was replaced by a rabbit antibody raised against acetylated histone H4 [31]; no signal would be expected since the histones are localised in the germinal vesicle, away from the cytoplasmic Vg1 mRNA. The result obtained showed that only 10% of these control oocytes (3/30) had a localised signal at the vegetal pole which was far fainter than that with the rabbit anti-XStau1 antibody but nonetheless detectable.

## Conclusions

We have shown that an RNA-protein complex can be detected with good spatial resolution using a variation of the ISH-PLA technique: rISH-PLA. There are a number of advantages of the rISH-PLA technique over traditional RNA immunoprecipitation assays used for studying RNA-protein interactions such as as RIP and CLIP. Principally these relate to its application to mixed cell populations and ease of use. ISH-PLA can provide information on where RNA-protein complexes are localised and allows the identification of a given RNA-protein interaction at subcellular and single cell resolution, thus avoiding issues associated with heterogenous cell populations from which conventional RNA-protein assays suffer. The detailed examination of RBP interactions with a specific RNA has always been a technically demanding series of experiments. Thus the potential to identify interactions at a cellular level in a relatively straightforward process, that can be adopted by any laboratory which routinely uses either immunohistochemistry or in situ hybridisation promises significant advantages for those investigating the role of such complexes in gene regulation, a rapidly growing area of research.

However, the rISH-PLA technique does appear to have at least some of the false positive issues associated with immunoprecipitation techniques. The results showed that 10% of the oocytes (3 out of 30) had a faint but visible localised signal at the vegetal pole in the control antibody samples. The reason for this is unclear but one possibility is that the anti-mouse PLA antibodies are anchored to the mouse anti-biotin antibody and that the anti-rabbit PLA antibody is not completely washed away due to permeability issues associated with the high lipid content of the oocytes in the vegetal region. Should this occur, then at the ligation step there is stochastic chance of the ligation of the two PLA antibodies interacting (although only one of them is anchored at this target) causing a signal at the vegetal pole. It will be interesting to discover whether this is an oocyte-specific problem caused by the high lipid content. Also it was important to have detailed information about the RNA-protein complex in order to design the probe efficiently. A lack of such information would not preclude the assay being applied but which probe to use would have to be determined empirically.

There are many RNA-protein interactions occurring that are of interest both for basic biology and biomedical research that may be advanced by rISH-PLA. Recently it has been found that more than 800 RBPs, which are often highly conserved across organisms, have been implicated as having crucial roles in several types of cancer [32]. RNA binding proteins are also critical in long non-coding RNAs [33,34], RNA processing events for miRNAs [35–37], tissue specific splicing events [35,38,39] and even transcriptional control [40].

The development of the rISH-PLA assay for RNA analysis provides a powerful tool to advance our understanding of the role of RNA binding proteins in gene regulation at the post-transcriptional level and their role in wider cellular activity. This method can potentially be used in other organisms, however crucial to the technique is the design of the LNA probe. This requires knowledge of the primary sequence of the target mRNA for secondary and tertiary structure prediction. Therefore its utility will be limited to organisms (such as *Drosophila*, Zebrafish and *Xenopus*) that have good sequence data.

## Supporting Information

S1 Text rISH-PLA Full Protocol. The full in detail step-by-step method for rISH-PLA is provided in the protocol shown in supplementary information.
(DOCX)
