# Supplementary material for: RNA Whole-Mount In situ Hybridisation Proximity Ligation Assay (rISH-PLA), an Assay for Detecting RNA-Protein Complexes in Intact Cells
Source: PLoS One. 2016 Jan 29;11(1):e0147967. doi: 10.1371/journal.pone.0147967 (PMC4732756; doi:10.1371/journal.pone.0147967)
Supplement: S1 Text — The full in detail step-by-step method for rISH-PLA is provided in the protocol shown in supplementary information. (DOCX) [file pone.0147967.s001.docx]

**S1: rISH-PLA Full Protocol.**

**Day 1**

1. Para-formaldehyde fixed *Xenopus laevis* oocytes were transferred to 1.5 ml Eppendorf tubes
2. The fixed oocytes were rehydrated by washing at room temperature (RT) flat on the bench with:

1 ml of 75 % Methanol in 1xPBST for 5 min

1 ml of 50 % Methanol in 1xPBST for 5 min

1 ml of 25 % Methanol in 1xPBST for 5 min

1 ml of 1xPBST for 5 min

1. The oocytes were washed with 1 ml of 0.1 M triethanolamine for 5 min at RT.
2. The oocytes were then washed with 1 ml of 0.1 M triethanolamine with 3 μl acetic anhydride added for 5 min at RT.
3. A further 3 μl acetic anhydride were added and the oocytes incubated for a further 5 min.
4. The oocytes were then washed 2 times with 1 ml PBST for 5 min in RT and then

2 times with 1 ml of Hybridisation Buffer for 2 min at RT.

1. The hybridisation buffer was replaced with a fresh 1 ml aliquot and incubated in a heating block on its side for: 30 min at 70 ⁰C and then 5 hours and 30 min at 60 ⁰C
2. The oocytes were then incubated overnight (O/N) at 60 ⁰C with 200 nM of LNA probe in a fresh 1 ml aliquot of hybridisation buffer.

**Day 2**

1. The oocytes were washed in a heating block placed on its side with the following pre-warmed at 60 ⁰C solutions: 1 ml of 50 % deionised formamide/5 x SSC for 10 min

1 ml of 25 % deionised formamide/2 x SSC for 10 min

1 ml of 12.5 % deionised formamide/2 x SSC for 10 min

1 ml 2 x SSC/0.1 % Tween20 for 10 min

1 ml 0.2 x SSC/0.1 % Tween20 for 30 min

1. Then the oocytes were washed at RT with gentle rocking:

3 times with 1 ml 1xPBST for 5 min

1 time with 1 ml of MAB for 10 min

1 time with 1 ml MAB/2 % Block for 5 hours

1. The oocytes were then transferred to a 250 μl PCR tube that had parafilm in the bottom of it so that it was limited to hold a volume of 100 μl in order to use minimum amount of the PLA solutions.
2. The oocytes were then blocked with 100 μl of PLA kit blocking solution.
3. The blocking solution was removed and the primary antibodies added diluted in the Antibody Diluent provided by the PLA kit (1:100) and the oocytes incubated O/N at 4⁰C with gentle rocking.

**Day 3**

1. The oocytes were transferred back into a 1.5 ml Eppendorf tube.
2. Excess unbound primary antibodies were removed by washing 3 times for 5 min at RT on a rocker with 1ml of 1 x Wash Buffer A (provided by the kit).
3. The oocytes were transferred again into parafilm stuffed PCR tube and then incubated with 20 μl PLA probe Minus, 20 μl PLA probe Plus and 60 μl of antibody diluent (all provided by the kit) for 2 hours and 30 min at 37 ⁰C with gentle rocking.
4. The oocytes were then transferred back into a 1.5 ml Eppendorf tube.
5. Excess unbound secondary antibodies were removed by washing 3 times for 5 min at RT on a rocker with 1ml of 1 x Wash Buffer A (provided by the kit).
6. The oocytes were then incubated with the ligation solution (20 μl of 5 x ligation stock, 77.5 μl of Sigma H_2_O (SH_2_O) and 2.5 μl Ligase) provided by the kit for 1 hour at 37 ⁰C with gentle rocking.
7. The oocytes were transferred back into a 1.5 ml Eppendorf tube.
8. Excess unbound ligation solution was removed by washing 3 times for 5 min at RT on a rocker with 1ml of 1 x Wash Buffer A.
9. The oocytes were then incubated with the amplification solution (20 μl of 5 x amplification stock, 78.7 μl of SH_2_O and 1.3 μl of polymerase) provided by the kit for 3 hours and 20 min at 37 ⁰C with gentle rocking covered with foil.
10. The oocytes were transferred back into a 1.5 ml Eppendorf tube.
11. The amplification solution was removed and the oocytes washed 2 times with 1 ml of 1 x Wash Buffer B (provided by the kit) for 10 min at RT.
12. A further 2 min wash with 0.01 x Wash Buffer B was performed prior to orientation of the oocytes on the glass slide and visualisation by confocal microscopy.

**NOTE: Vortex all PLA kit Solutions before use**

**During and after the amplification reaction always cover with foil.**
